# A Case of Cervical Necrotising Fasciitis Precipitated by Hypopharyngeal Squamous Cell Carcinoma

**DOI:** 10.7759/cureus.109391

**Published:** 2026-05-21

**Authors:** Saffron G O'Neill, Tiarnan Mellon, Gabrielle Croker, Clare Batchelor, Stephanie Flukes

**Affiliations:** 1 Department of Otolaryngology Head and Neck Surgery, Fiona Stanley Hospital, Perth, AUS; 2 Department of General Surgery, Royal Perth Hospital, Perth, AUS; 3 Department of Plastic and Reconstructive Surgery, Fiona Stanley Hospital, Perth, AUS; 4 Division of Surgery, University of Western Australia, Perth, AUS

**Keywords:** cervical necrotising fasciitis, head and neck pathologies, hypopharyngeal cancer, necrotising fasciitis, necrotising fasciitis (nf)

## Abstract

Cervical necrotising fasciitis (CNF) is a rare but life-threatening soft tissue infection of the head and neck, associated with high morbidity and mortality despite advances in disease detection and antimicrobial therapy. It most commonly arises from odontogenic or pharyngeal sources, and presentation secondary to underlying head and neck malignancy is uncommon.

We report the case of a 54-year-old woman who presented with rapidly progressive anterior neck swelling, subcutaneous emphysema, and septic shock. Cross-sectional imaging demonstrated extensive gas-forming infection involving multiple cervical fascial planes with mediastinal extension. The patient underwent radical surgical debridement and thoracic washout. Intraoperative laryngoscopy revealed ulcerated mucosa in the left pyriform fossa with a suspected mucosal perforation, with biopsy confirming moderately differentiated squamous cell carcinoma (SCC). The patient stabilised with broad-spectrum antimicrobial therapy and intensive care support, but in light of the cancer diagnosis, her family decided to withdraw active management, and the patient died five days following admission.

This case represents a rare presentation of CNF arising secondary to an occult oropharyngeal SCC. Tumour-associated mucosal breach and local tissue invasion likely facilitated bacterial spread into deep cervical spaces. The presence of mediastinal extension, septic shock, and underlying malignancy contributed to the poor prognosis despite timely surgical intervention.

CNF may represent a first manifestation of head and neck malignancy. In the absence of an identifiable odontogenic or traumatic source, clinicians should maintain a high index of suspicion for underlying mucosal perforation. Early recognition, prompt surgical management, and multidisciplinary care are the cornerstones in the management of CNF, although outcomes remain poor in advanced disease.

## Introduction

Necrotising fasciitis is a fulminant, rapidly progressive soft tissue infection characterised by fascial plane involvement, microvascular thrombosis, and subsequent tissue necrosis. Despite advances in critical care and antimicrobial therapy, necrotising fasciitis continues to carry substantial morbidity and mortality [1].

The pathophysiology of necrotising fasciitis is characterised by a self-perpetuating cycle of bacterial invasion, host immune dysregulation, and progressive tissue destruction. Following disruption of the mucocutaneous barrier, infection spreads rapidly along planes of least resistance, typically the relatively avascular superficial fascial layers. Bacterial toxin release drives activation of a coagulation cascade, creating a hypercoagulable environment within the tissues’ capillaries and perforating vessels. The resulting tissue ischaemia impairs immune cell infiltration and antibiotic delivery to the affected site. In cases of necrotising fasciitis, the extent of tissue damage is often disproportionate to the external appearance [2].

The abdomen, perineum, and extremities are the most commonly affected sites, with necrotising fasciitis typically occurring here following minor trauma, surgery, or mucocutaneous disruption. In contrast, necrotising fasciitis involving the head and neck is rare, accounting for less than 10% of cases. Cervical necrotising fasciitis (CNF) is seldom reported in scientific literature, with available articles largely limited to case reports and small institutional reviews [1,3].

Infection is most commonly polymicrobial in nature and arises from odontogenic or pharyngeal sources - implicated pathogens include Streptococcus spp., *Staphylococcus aureus*, and oral anaerobes [1]. Survival relies on early surgical debridement, combined with broad-spectrum intravenous antibiotic therapy and robust supportive care [4].

Several host-related factors are recognised as predisposing to the development of CNF. Diabetes mellitus is the most consistently implicated comorbidity, reported in up to 43% of cases in larger series, with immunosuppression, chronic renal failure, and malignancy also implicated [5]. These conditions are known to impair local immune defenses and microvascular perfusion, facilitating bacterial invasion of fascial planes and blunting the early inflammatory response.

Hypopharyngeal squamous cell carcinoma (SCC) accounts for approximately 3% of head and neck malignancies, with the pyriform sinus representing the most common subsite. The majority of hypopharyngeal SCC cases are diagnosed at a locoregionally advanced stage, a pattern largely attributable to the anatomical characteristics of the hypopharynx. Early mucosal tumours in the pyriform sinuses produce minimal localising symptoms; hence, the majority of hypopharyngeal lesions can grow substantially to the point of ulceration before generating red flag symptoms such as odynophagia, dysphagia, or hoarseness [6].

While CNF in itself is uncommon, CNF as a first presentation of an occult malignancy is exceedingly rare and presents a diagnostically challenging scenario. Very few reported cases exist in the English-language literature describing CNF as a presenting feature of hypopharyngeal malignancy, underscoring the educational value of this case. We will discuss a case of CNF arising secondary to a perforating hypopharyngeal mass, with discussion pertaining to the presentation, diagnosis, and management.

## Case presentation

A 54-year-old woman was brought in an ambulance to the emergency department after being found unresponsive in her home by her family. This had been preceded by two days of progressive anterior neck pain and swelling. On arrival, she had a large anterior neck swelling with mottled, discoloured skin, and extensive subcutaneous emphysema (Figure 1). She rapidly became hypoxic, tachycardic, and hypotensive and was intubated, with some difficulty.

**Figure 1:**
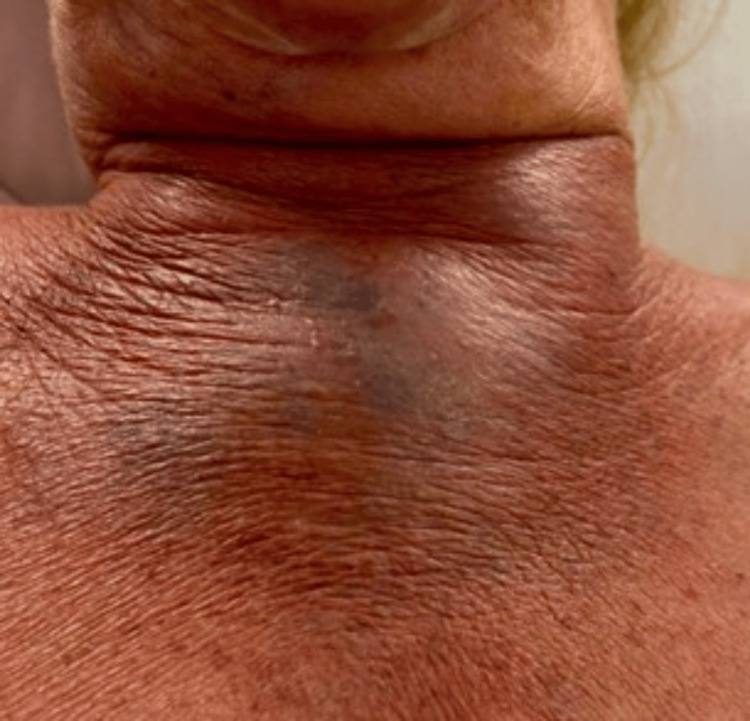
Anterior neck and superior chest following extensive debridement of necrotic tissue.

Cross-sectional imaging was suggestive of necrotising fasciitis with multicompartmental involvement in the neck, including bilateral anterior cervical spaces, visceral space including left haemithyroid retropharyngeal space, and left carotid space, with mediastinal extension. See below Figures 2-4 - CT neck and chest with IV contrast - depicting extensive gas-forming infection within the soft tissues of the neck and mediastinum.

**Figure 2:**
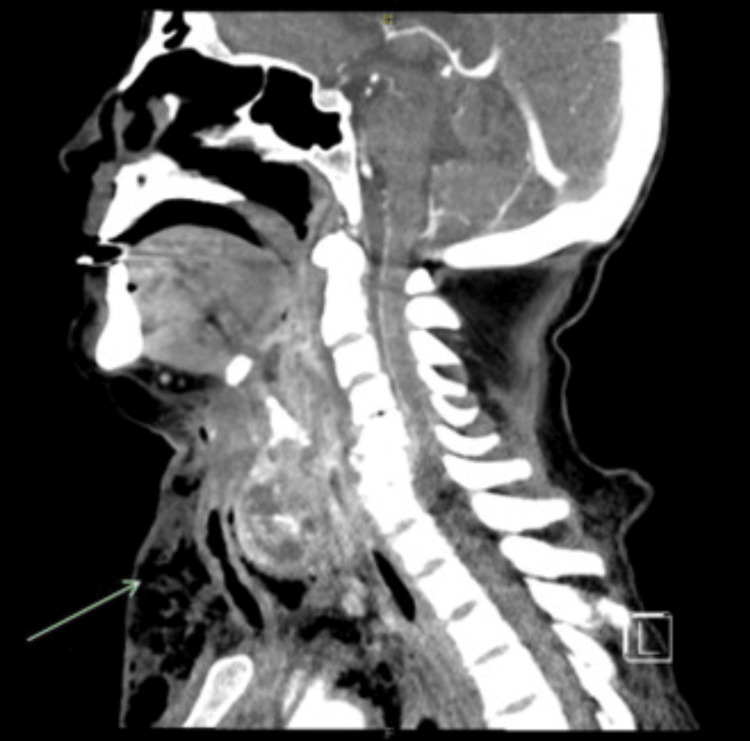
CT imaging - sagittal view with an arrow indicating towards gas in the anterior neck and mediastinum. CT: computed tomography.

**Figure 3:**
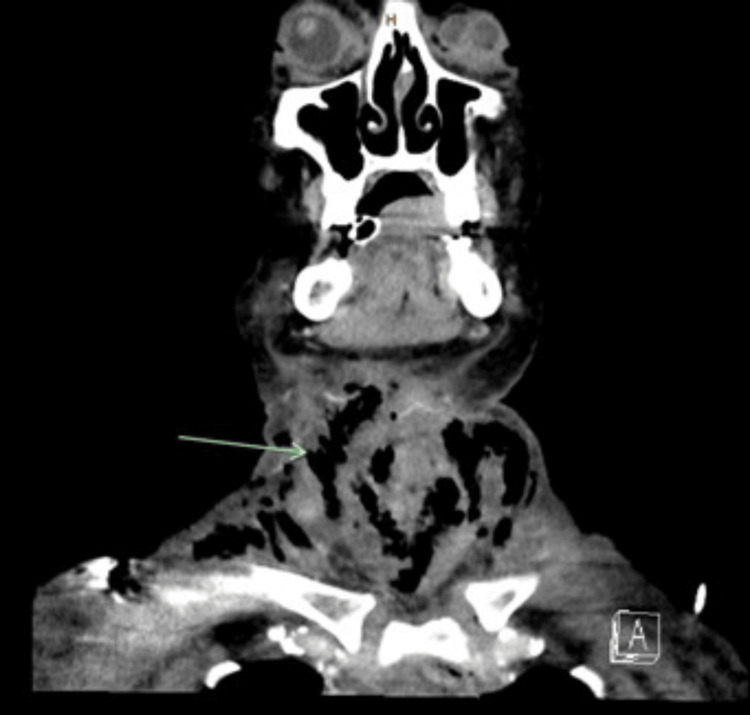
CT imaging - coronal view with an arrow indicating towards gas in the anterior neck and mediastinum. CT: computed tomography.

**Figure 4:**
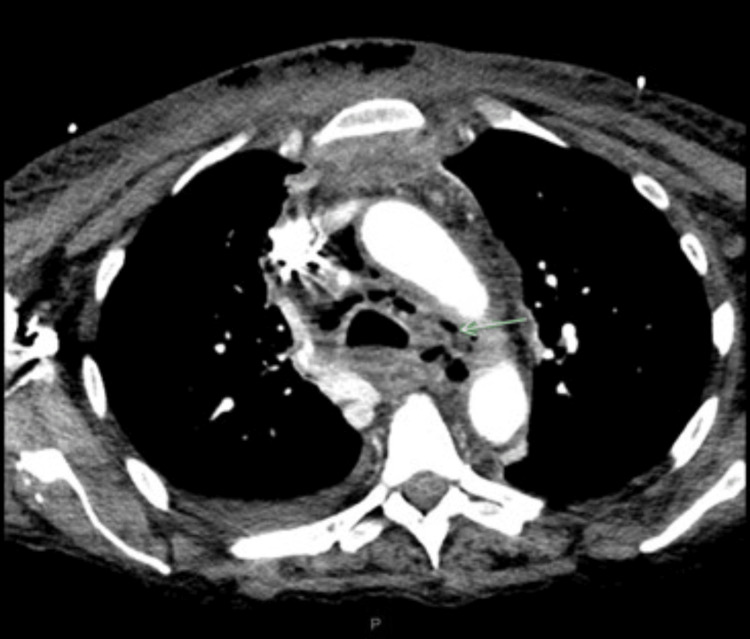
CT imaging - axial view of a collection with an arrow indicating towards gas bubbles within the mediastinum. CT: computed tomography.

The patient was commenced on empirical broad spectrum antibiotic therapy (Meropenem 1 g TDS, Vancomycin 1.5 g BD, and Clindamycin 600 mg TDS) and proceeded to theatre for emergent debridement. The procedure involved resection of cervical fasciocutaneous tissue and left hemithyroidectomy, combined with a right posterolateral thoracotomy and washout of descending mediastinitis. Intraoperative findings revealed extensive necrotising soft tissue infection involving anterior cervical skin and subcutaneous tissue, bilateral sternocleidomastoid, and strap muscles, which tracked into the anterior mediastinum.

Direct laryngoscopy identified an ulcerated area of pharyngeal mucosa in the left pyriform fossa with suspected mucosal perforation. Subsequent biopsy and histopathological analysis revealed moderately differentiated SCC. There was no obvious dental abscess or other area of potential introduction of infection elsewhere. At the conclusion of the case, the anterior neck was left open (Figure 5), with plans for further washout and reconstruction at a later point. Intra-operative fluid samples obtained from the neck showed no growth (thought due to early initiation of antibiotic therapy), and those from the mediastinum grew *Streptococcus anginosus*.

**Figure 5:**
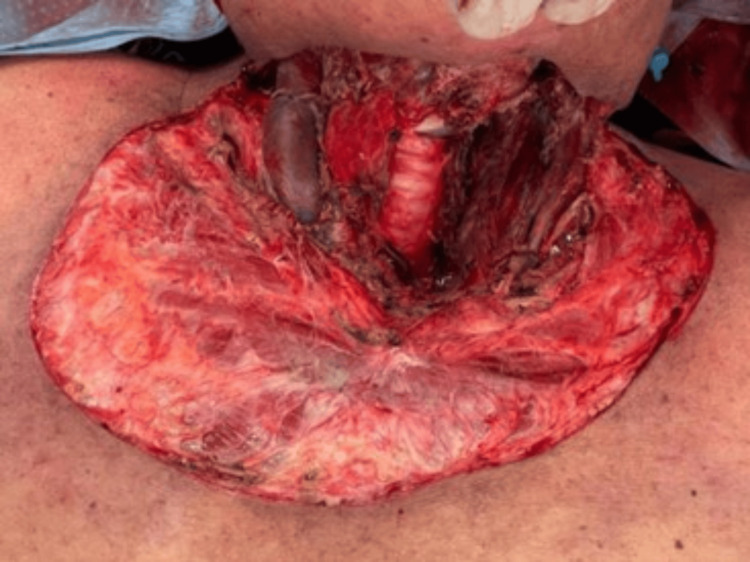
Anterior neck and superior chest following extensive debridement of necrotic tissue.

The patient was cared for in an intensive care setting in the following days and remained intubated and sedated. She had a persistent vasopressor requirement throughout due to ongoing septic shock, complicated further by new atrial fibrillation, which was managed effectively with amiodarone infusion.

Wound care was complex and required specialty input from colleagues in plastics and reconstructive surgery. The wounds were regularly examined for signs of advancing infection or tissue necrosis, and they were cleaned and dressed under sterile conditions using a soft silicone wound contact layer to open areas (which included large vessels and cervical viscera), normal saline burns gauze superficially, and a transparent film dressing to seal. Emphasis was placed on the importance of ensuring no pressure was applied to the area. Hyperbaric oxygen therapy was considered as an adjunct to the above for wound management; however, given the relative contraindication due to underlying piriform fossa SCC, the decision was made not to proceed.

The patient remained stable on this regimen for several days. Following histopathological confirmation of hypopharyngeal SCC, a discussion was held with the patients’ family regarding goals of care. Treatment of the SCC would require an extended laryngopharyngectomy, and the likelihood of successful outcome was felt to be low. The family elected to pursue a palliative treatment course and withdrew active care. The patient died on day 5 of hospital admission.

## Discussion

This case illustrates a rare and fulminant presentation of CNF arising secondary to an underlying oropharyngeal SCC. CNF remains an uncommon but highly lethal form of soft tissue infection of the head and neck, with contemporary literature consistently reporting mortality rates of approximately 25-40%, particularly in the presence of septic shock or mediastinal extension [1,3,4,7]. Outcomes remain poor despite advances in antimicrobial therapy.

The association between CNF and head and neck malignancy is exceedingly rare. Gunaratne et al.’s systematic review of 1,235 cases identified malignancy as a contributing factor in only a small minority of patients [1]. Proposed mechanisms of infection in these cases include tumour-associated mucosal ulceration and direct invasion of adjacent soft tissues, factored with an immunocompromised state. It is suggested that these factors contribute to the translocation of oral flora into cervical fascial planes [4,7]. In the case discussed here, no odontogenic or traumatic source was identified, and the ulcerated left pyriform fossa SCC was considered the most likely source of infection.

CNF is most commonly polymicrobial, involving a mixture of aerobic and anaerobic organisms derived from the oral cavity and upper aerodigestive tract [1,3,7]. The isolation of *Streptococcus Anginosus* in this case is consistent with previously documented microbiological profiles of deep neck space infection and descending mediastinitis [8].

Current guidance for the management of CNF emphasises early recognition, airway protection, immediate initiation of broad-spectrum parenteral antibiotics, and urgent surgical debridement, with early multidisciplinary input, including ENT, cardiothoracics, intensive care, infectious diseases, and plastic surgeons highlighted as critical to improving outcomes [1,3,4,7,9]. Despite early surgical intervention and appropriate antimicrobial therapy in this case, the presence of septic shock, mediastinal extension, and underlying malignancy conferred a poor prognosis from the point of presentation.

The Laboratory Risk Indicator for Necrotising Fasciitis (LRINEC) score was developed as a screening tool to help distinguish necotising fasciitis form other severe soft tissue infections. The score is derived from routine haematological and biochemical parameters, including CRP, white cell count, haemoglobin, sodium, creatinine, and glucose. A score of ≥6 has been associated with a positive predictive value of 92% in the original derivation study [10], with Kim et al. determining that in a head and neck population, the score had a sensitivity of 79.1% and specificity of 96% at the same cut off [11]. In the present case, the combination of clinical findings and CT evidence of multi-compartmental gas-forming infection was sufficient for diagnosis, and therefore, the LRINEC score was not calculated.

Descending necrotising mediastinitis represents a potentially catastrophic complication of CNF, occurring when infection tracks inferiorly through fascial planes into the mediastinum. Endo et al. proposed a now widely used classification: type I (localised to the upper mediastinum), type IIa (diffuse anterior mediastinitis), and type IIb (total mediastinal involvement), with types IIa/IIb associated with significantly higher mortality [12]. Prado-Calleros et al. determined that 17-21% of cases of CNF proceeded to develop descending necrotising mediastinitis, and that this carried a combined mortality of 26-41% [13]. In this case, the present descending necrotising mediastinitis fell under Endo class IIb, with both anterior and posterior mediastinal involvement, thus necessitating need for drainage via posterolateral thoracotomy.

The role of hyperbaric oxygen therapy (HBOT) in CNF remains controversial. While some studies suggest potential benefit in necrotising soft tissue infections, evidence specific to CNF is limited, and its use may be constrained by patient instability and oncological considerations in the setting of active head and neck malignancy [4,9,14]. The decision not to offer HBOT in this case was therefore consistent with recommendations of the literature at this time.

## Conclusions

CNF remains a rare but devastating condition with high mortality. This case highlights an unusual presentation of CNF arising secondary to an occult hypopharyngeal malignancy, with infection likely facilitated by tumour-mediated mucosal breach. In patients presenting with rapidly progressive cervical infection without an obvious odontogenic or traumatic source, early endoscopic evaluation to exclude occult malignancy or perforation should be considered. Despite prompt surgical debridement, broad-spectrum antimicrobial use and coordinated multidisciplinary intervention, prognosis remains poor.
